# Emotion Regulation and Attitudes Toward Conflict in Colombia: Effects of Reappraisal Training on Negative Emotions and Support for Conciliatory and Aggressive Statements

**DOI:** 10.3389/fpsyg.2019.00908

**Published:** 2019-04-24

**Authors:** Camilo Hurtado-Parrado, Myriam Sierra-Puentes, Mohammed El Hazzouri, Alexandra Morales, Diana Gutiérrez-Villamarín, Laura Velásquez, Andrea Correa-Chica, Juan Carlos Rincón, Karen Henao, Juan Gabriel Castañeda, Wilson López-López

**Affiliations:** ^1^Department of Psychology, Troy University, Troy, AL, United States; ^2^Faculty of Psychology, Fundación Universitaria Konrad Lorenz, Bogotá, Colombia; ^3^Mount Royal University, Bissett School of Business, Calgary, AB, Canada; ^4^Pontificia Universidad Javeriana, Department of Psychology, Bogotá, Colombia; ^5^Universidade de Santiago de Compostela, Departamento de Psicoloxía Social, Básica e Metodoloxía, Facultade de Psicoloxía, Santiago de Compostela, Spain; ^6^Corporación Universitaria Minuto de Dios, Dirección de Investigaciones, Bogotá, Colombia

**Keywords:** emotion regulation, cognitive reappraisal, conciliation, aggression, peace, intractable conflict, Colombia, FARC-EP

## Abstract

Control of negative emotions (e.g., anger and fear) by political cues perpetuate intractable conflict by mobilizing public support for aggressive actions. Halperin et al. (2013) found that reappraisal – an adaptive form of emotion regulation – decreased negative emotions triggered by anger-inducing information related to the Israeli–Palestinian conflict, and increased support for conciliatory statements. We tested these effects in the context of the conflict between the Colombian government and the Fuerzas Armadas Revolucionarias de Colombia-Ejército del Pueblo (FARC-EP). Reappraisal training reduced negative emotions produced by a presentation that illustrated FARC’s violent actions, and increased support for conciliatory statements (with overall moderate effect magnitudes). We also found that negative emotions mediated the effects of reappraisal on the support for aggressive and conciliatory statements. These findings indicate a high degree of generality of the phenomena, especially considering the differences between the Israeli–Palestinian conflict and the Colombian conflict. Our findings also show promise for replicating these effects on other types of intergroup conflicts and guiding effective public policy.

## Introduction

Violent intractable conflicts occupy a central social and political position in the affected societies and require immense material and psychological investment (Kriesberg, 1993; Bar-Tal, 1998; Gross et al., 2013). Individuals living in areas of intractable conflict frequently experience high intensity negative emotions, such as anger, fear, despair, and hatred, irrespective of how directly the events inherent to those conflicts affect them (Halperin, 2014; Rosler et al., 2015). The grief over the loss of known or unknown combatants or civilian casualties, fear from being hurt or having a friend or relative hurt, and despair over failed attempts to achieve peace are the type of intense emotions experienced by members of societies affected by intractable conflict. In this context, political cues gain considerable control over these negative emotions, which in turn, seem to perpetuate the continuation of conflicts by mobilizing public support for aggressive actions, and hindering progress toward conflict resolution (Gross et al., 2013; Halperin et al., 2013; Halperin, 2014).

If negative emotions related to conflict limit the attempts toward a conflict’s peaceful resolution, it seems plausible that efforts to decrease such emotions could result in important outcomes. Research that has extended the field of emotion regulation (Gross, 2013, 2014) to the study of conflict resolution has provided evidence in support of this notion (Gross et al., 2013; Halperin et al., 2013; Halperin, 2014; Halperin and Pliskin, 2015; Rosler et al., 2015; Čehajić-Clancy et al., 2016; Gutentag et al., 2016).

### Emotion Regulation and Intractable Conflict: The Effects of Cognitive Reappraisal

Emotion regulation has been defined as the processes by which individuals influence the intensity and valence of their emotions, when they have them, and how they are expressed (Gross, 2014). Individuals implement a wide range of emotion regulation strategies during their day-to-day aversive interactions, including distraction, rumination, wishful thinking, and social sharing (Páez et al., 2013). However, they are not equally adaptive in terms of effectiveness to decrease subjective distress and/or physiological arousal, while maintaining the individual’s ability to pursue meaningful short- and long-term personal and interpersonal goals (Gross, 2014). Cognitive reappraisal is an emotion regulation strategy aimed at reducing or increasing emotional responses by reinterpreting the meaning of the triggering situation (Gross, 2002). This reappraisal process therefore entails detecting and assessing the significance of the environment for wellbeing (Frijda, 2009) - e.g., perceiving the positive aspects of the triggering event, changing its personal relevance, or imagining better or worse outcomes. Over the last decade, mounting research has shown that reappraisal importantly improves physical and psychological well-being (Gross and John, 2003; Webb et al., 2012; Gross, 2014; Hu et al., 2014; Picó-Pérez et al., 2017). However, only recently it has been suggested that it also has a positive role in larger scale phenomena, such as intractable conflicts.

In the context of the Israeli–Palestinian conflict, Halperin and Gross (2011) found a positive correlation between the implementation of reappraisal and support for providing humanitarian aid to Palestinian citizens by Israeli citizens. More recently, direct evidence of the causal role of emotion regulation on changes to political attitudes in intractable conflicts was obtained. Halperin et al. (2013) tested if reappraisal training could reduce negative intergroup emotions and, in doing so, could decrease support for aggressive actions and increase conciliatory attitudes toward conflict-related events. Israeli participants (college students) first received either reappraisal training or were assigned to a control condition, which in both cases was followed by a presentation of anger-inducing information related to the Israeli–Palestinian conflict (texts, pictures, and music). After the presentation, the participants indicated the extent to which they felt positive and negative emotions, together with their degree of support for different statements reflecting conciliatory policies (e.g., “Regardless of the security situation, Israel needs to transfer food, and medication to Gaza residents”) and aggressive policies (e.g., “If the Israeli Defense Forces detects a terrorist in a building full of civilians, Israel should bomb the building even if most of the civilians will most likely be killed”) toward Palestinians in the Gaza strip. Halperin et al. (2013) found that (a) participants in the reappraisal condition reported lower levels of negative emotions (e.g., anger) compared with control participants; (b) participants in the reappraisal condition expressed more support for conciliatory statements and less support for aggressive statements than participants in the control condition; (c) negative emotions were positively associated with support for aggressive statements and negatively associated with support for conciliatory statements; and (d) negative emotions, specially anger, mediated the effect of reappraisal on support for conciliatory and aggressive statements. In our research, we aimed to reproduce these findings in the Colombian context.

The present study was conceived by the time major efforts to end the more than five-decade Colombian conflict between the government and the FARC-EP (Revolutionary Armed Forces of Colombia – Army of the People) were being implemented. Also, with consideration of the potential extension of the findings reported by Halperin et al. (2013) regarding the role of emotion regulation on changing attitudes toward conciliatory and aggressive policies in intractable conflicts.

### The Colombian Government-FARC Conflict and the Peace Accord

Fuerzas Armadas Revolucionarias de Colombia (FARC) was officially founded in 1964. It is one of the multiple expressions of agrarian struggles that emerged in Colombian rural areas during the 1930s, and the bipartisan-fueled violence that erupted during the 1950s. FARC shared with other guerrillas the influence of the ideological and political climate of the Cold War and the Cuban Revolution (Thomson, 2011; Centro Nacional de Memoria Histórica, 2013).

FARC proclaimed in the 1980s that their goal was to overthrow the Colombian government. During the same decade, other armed actors emerged, including right-wing paramilitary groups, which had ties to the army, political figures, drug cartels, and large landowners. By the end of the decade, the conflict escalated, including a dramatic upsurge in violent actions affecting the civilian population (e.g., terrorist acts, kidnapping, massacres, landmines, forced recruitment, forced displacement, disappearances, and selective executions; Abello-Llanos et al., 2009; Grajales, 2011).

Drug trafficking became a major funding source for both paramilitary groups and the FARC during the following decades, which resulted in their strengthening. The Colombian state forces (police and army) also grew substantially as response to the consolidation of the other actors. The end result was a modernized and exacerbated conflict, which further affected the civilian population – including frequent human rights’ violations. The estimated number of victims surpassed six million (Human Rights Watch, 2015; Unidad para las Victimas, 2017), including millions of internally displaced and more than 200,000 deaths (near 80% civilians; Historical Memory Group, 2016).

The first step toward the termination of the Government-FARC conflict was the signing of the “General Accord for the Termination of the Conflict and the Construction of a Stable and Lasting Peace” on August 26, 2012 in Havana, Cuba. After 4 years of negotiations, the parties signed on June 23, 2016 the 6-point accord on the end of the conflict, which included agreements related to an integral rural reform, political participation, a bilateral and definite ceasefire and cessation of hostilities, security guarantees for human-rights defenders and social movements, a solution to the problem of illicit drugs, victims’ reparation and compensation, and mechanisms for implementation and verification of the accord (Colombian Government, 2016a; Amnesty International, 2017).

In addition, the parties agreed on a national referendum (plebiscite) as a mechanism for ratification of the signed peace accord, which took place on October 2, 2016. With a 37.4% turnout, 50.2% of Colombians voted against it, and 49.8% voted in favor (Registraduria Nacional del Estado Civil, 2016). This unexpected result obligated the Colombian government and FARC to revise the agreement, this time with the participation of the opposition. The amended version of the agreement was approved by the Colombian Senate and the House of Representatives on November 29 and 30, 2016 (Colombian Government, 2016b).

Emotional factors had an important role in the difficulties that the peace process encountered – especially the results of the national referendum – which continue to be key to the success of ongoing processes of forgiveness and reconciliation (López-López et al., 2013, 2018; Cortés et al., 2015). The success of the controversial campaign ran by the opposition party (Centro Democrático) against the accord supported this notion. Reports indicate that one of its main strategies was triggering negative emotions in the voters, such as anger and fear, by distorting some of the contents of the accord regarding impunity for the FARC members and the negative economic and political implications of accepting the accord (El Espectador, 2016; El País, 2016).

### Overview of the Study

The present study aimed to test whether the effects of cognitive reappraisal training reported by Halperin et al. (2013) could be reproduced in the context of the Colombian government-FARC conflict and the efforts of these actors to end the conflict. The experiment was conducted during the week before the national referendum on the acceptance of the peace agreement took place (October 2, 2016). The methodology of Halperin et al.’s (2013) study was adapted to systematically replicate, as close as possible, the conditions of their experiments (e.g., selection of relevant images and texts for the anger-inducing presentation, measurement of negative emotions, and selection of the conciliatory and aggressive statements toward FARC). Analogous to Halperin et al. (2013) study, college students received cognitive reappraisal training, or a control condition, followed by a presentation of anger-inducing information related to FARC’s violent actions during the conflict. Immediately after the presentation, the participants indicated the extent to which they felt positive and negative emotions. They were also asked to rate their degree of support for different conciliatory or aggressive statements toward FARC, which were prepared by taking excerpts from the peace accord already signed between the involved parts (to be voted on the national referendum; Colombian Government, 2016a) or quotes of public statements made by political opponents to that peace agreement (published in national newspapers). Based on Halperin et al.’s (2013) findings, we predicted that reappraisal training would have resulted in (a) lower levels of negative emotions triggered by anger-inducing information related to FARC’s violent actions, and (b) more support for conciliatory statements and less for aggressive statements. In addition, we expected that (c) negative emotions, including anger, would mediate the effect of reappraisal on support for conciliatory and aggressive statements.

## Materials and Methods

### Participants

One-hundred eight college students voluntarily participated in the study (69 women and 39 men, *M*_age_ = 20.3 years; *SD*_age_ = 1.79), which were recruited from the following undergraduate psychology programs in Bogotá (Colombia): Konrad Lorenz Fundación Universitaria (*n* = 24), Universidad San Buenaventura (*n* = 24), Pontificia Universidad Javeriana (*n* = 22), and Corporación Universitaria Minuto de Dios – Soacha (*n* = 38). These institutions were selected to capture the wide range of socioeconomic statuses that characterize the Colombian population. Students received a refreshment at the end of the study in return for their participation.

This study was carried out in accordance with the ethical guidelines and recommendations of the American Psychological Association – APA (2002) and the Colombian Board of Psychologists (2016). All subjects gave written informed consent in accordance with the Declaration of Helsinki. The protocol was approved by the Research and Ethics Committee at Konrad Lorenz Fundación Universitaria.

### Procedure

Students were invited to the laboratory to participate in a study related to human emotion. Initially, they completed a trauma inventory (Bremner et al., 2007) aimed at screening their direct experience with the events that were portrayed in subsequent phases of the experiment (e.g., they were asked if they had witnessed a murder or violent acts). As per recommendation of the ethics committee that oversaw the study, participants who reported any of the traumatic experiences listed in the inventory did not continue to further stages of the experiment (*n* = 31, different from the 108 participants of the study). The different phases of the experiment are described below, which were applied in a single session (approximately 15 min). The participants were individually tested.

#### Reappraisal Training

Students were randomly assigned to a reappraisal-training condition (*n* = 54) or a control condition (*n* = 54). The protocol for the reappraisal intervention replicated that reported by Halperin et al. (2013), who adapted it from that implemented by Richards and Gross (2000). Each participant was exposed to his/her corresponding condition individually. Using a Microsoft PowerPoint^®^presentation (available on request) projected in a computer screen, the participant observed five anger-inducing pictures related to the Colombian conflict (used from an ongoing project aimed at validating the emotional properties of conflict-related images), and was asked to “respond to them like scientists, objectively and analytically trying to think about them in a cold and detached manner” (Halperin et al., 2013, p. 2). The experimenter explained how to use the reappraisal technique in the presence of the first image, and the participant was asked to apply the technique in the presence of four additional pictures. During all the trials, the experimenter ensured that the participant applied the technique appropriately. Participants in the control condition were exposed to the same set of pictures, but they were only asked to respond to them naturally.

#### Presentation of Anger-Inducing Information

After the reappraisal or control manipulation, all participants watched a 4-min PowerPoint presentation that portrayed FARC’s violent acts (e.g., displacement, forced disappearance, and sexual violence – presentation available on request) with pictures, text, and music. This presentation was adapted to resemble, as close as possible, the presentation implemented by Halperin et al. (2013) – e.g., number of pictures, music, and length and content of the texts. Prior to the projection of the presentation, participants in the reappraisal condition were asked to apply the technique that they had learned in the previous phase, whereas those in the control group were reminded to respond naturally.

#### Negative/Positive Emotion Assessment

After the anger-inducing presentation, the participants indicated the degree to which they felt positive (interested, optimistic, strong, enthusiastic, proud, alert, inspired, determined, attentive, and active) and negative (angry, upset, guilty, scared, hostile, irritable, ashamed, nervous, jittery, and afraid) emotions using the Positive and Negative Affect Schedule (PANAS – Watson et al., 1988 – adapted to Spanish by Dufey and Fernández, 2012; α_positive_ = 0.78; α_negative_ = 0.87). Participants indicated to what extent they presently felt each emotion toward FARC using a Likert-style rating scale that ranged from 1 = “*very slightly or not at all*” to 5 = “*Extremely.*” Specific scores for each emotion and total scores for negative and positive emotions were analyzed. The sum of the scores on the corresponding individual emotions were used to produce *PANAS Negative* and *PANAS Positive scores* for each participant; accordingly, scores in each of these two measures could range between 10 and 50.

#### Support for Conciliatory and Aggressive Statements

In what was presented as a separate study on attitudes toward the Colombian conflict, the participants indicated their support of three statements reflecting conciliatory statements, and another three items reflecting aggressive statements. Conciliatory statements consisted of excerpts copied verbatim from the peace accord signed between FARC and the Colombian government (Colombian Government, 2016a), which was to be voted on a national referendum days after the experiment took place (e.g., “Reincorporation of the FARC forces to the civil life will be an integral and sustainable process, exceptional and transitory, that will take into account the interests of the community and FARC, including their members and their families, and which will be oriented toward the regional strengthening of the social tissue”). Aggressive statements consisted of verbatim public statements made by political opponents of that peace agreement (e.g., statements in national newspapers – “Although FARC announced 1 year ago a unilateral cease fire, they have not stopped dealing drugs or extorting. These are some of the criminal activities that they have never recognized, which affect the public order and the community” – Interview with Senator E. Macias by Diario del Huila, 2016). The Likert-style rating scale for the conciliatory and aggressive statements ranged from 1 = *highly oppose*, to 6 = *very much in favor.* Factor analysis revealed that there were indeed two factors, one measuring conciliatory statements (α_*conciliatory*_ = 0.65), and another measuring aggressive statements (α_aggressive_ = 0.68). See complete list of aggressive and conciliatory statements on Appendix 1 (see Supplementary Materials). Participants also completed the 33-item Marlowe–Crowne Social Desirability Scale (Crowne and Marlowe, 1960 – adapted to Spanish by Ferrando and Chico, 2000).

### Data Analyses

Halperin et al. (2013) tested the effects of reappraisal on overall negative and positive emotion scores (PANAS positive and PANAS negative) and scores on specific emotions (e.g., anger). Accordingly, we conducted one-tailed independent-samples *t* tests to assess directional effects of reappraisal on *PANAS negative* and *PANAS positive* and on each of the ten positive and ten negative emotions (e.g., based on Halperin et al.’s (2013) findings we expected significant decrements in negative emotions resulting from reappraisal training).

Also following Halperin et al. (2013) approach and findings, we (a) assessed whether reappraisal increased support for conciliatory statements and reduced support for aggressive statements using directional *t* tests; and (b) tested the mediating effects of negative emotions on support for aggressive and conciliatory statements using PROCESS procedure for SPSS with model 4 and 5000 bootstrap resamples (Hayes, 2013).

The potential effect of reappraisal on the scores of the Marlowe–Crowne Social Desirability Scale were assessed with a two-tailed independent-samples *t* test. Cohen-s’d effect-size calculations were conducted (when applicable).

## Results

### Effects of Reappraisal Training on Negative Emotions and Conciliatory and Aggressive Statements

A comparison of the overall negative emotions scores (PANAS negative) indicated that participants exposed to reappraisal training prior to the presentation of the anger-inducing information reported less intense negative emotions (*M* = 19.76, *SD* = 7,24) than participants in the control condition (*M* = 23.46, *SD* = 8,46), *t*(106) = 2.445, *p* = 0.008, Cohen’s *d* = 0.47. No significant differences in the overall scores of positive emotions (PANAS positive) were found between reappraisal (*M* = 24,81, *SD* = 6.80) and control (*M* = 24.31, *SD* = 5.87) groups; *t*(106) = 0.409, *p* = 0.342.

Similar to Halperin et al.’s (2013), we tested the effects of reappraisal on each individual positive and negative emotion using one-tailed independent-samples *t* tests. As shown in Table 1, reappraisal significantly reduced scores on anger, distress, fear, irritability, and uneasiness, with moderate-to-high effect magnitudes (Gignac and Szodorai, 2016), whereas it had no significant effect on participants’ reports of nervousness, shame, guiltiness, and hostility (*p*s > 0.115) or in any of the positive emotions assessed (*p*s > 0.145).

**Table 1:** One-tailed independent-samples *t* tests and effect sizes (Cohen’s d) for scores on specific negative emotions of participants in the reappraisal training (RT) and control condition (CC).

Angry	Upset	Fearful	Irritable	Uneasy	Scared
*M*_RT_ = 2.31	*M*_RT_ = 2.00	*M*_RT_ = 1.89	*M*_RT_ = 1.78	*M*_RT_ = 2.37	*M*_RT_ = 1.96
*M*_CC_ = 2.76	*M*_CC_ = 2.50	*M*_CC_ = 2.31	*M*_CC_ = 2.30	*M*_CC_ = 2.89	*M*_CC_ = 2.43
*SD*_RT_ = 1.24	*SD*_RT_ = 0.99	*SD*_RT_ = 1.08	*SD*_RT_ = 0.90	*SD*_RT_ = 1.39	*SD*_RT_ = 1.21
*SD*_CC_ = 1.24	*SD*_CC_ = 1.26	*SD*_CC_ = 1.23	*SD*_CC_ = 1.19	*SD*_CC_ = 1.25	*SD*_CC_ = 1.34
*t* = 1.859	*t* = 2.298	*t* = 1.919	*t* = 2.547	*t* = 2.034	*t* = 1.882
*df* = 106	*df* = 106	*df* = 106	*df* = 106	*df* = 106	*df* = 106
*p* = 0.033	*p* = 0.012	*p* = 0.029	*p* = 0.006	*p* = 0.022	*p* = 0.031
*d* = 0.36	*d* = 0.44	*d* = 0.37	*d* = 0.49	*d* = 0.39	*d* = 0.36

Participants in the reappraisal condition expressed more support for conciliatory statements (*M* = 4.88, *SD* = 0.93) when compared with participants in the control group (*M* = 4.56, *SD* = 0.96), *t*(106) = 1.735, *p* = 0.043, *d* = 0.33. The obtained Cohen’s d indicated a moderate effect of reappraisal on increasing support for conciliatory statements.

No significant differences between control (*M* = 3.42, *SD* = 1.14) and reappraisal groups (*M* = 3.36, *SD* = 1.32) were found regarding support for aggressive statements, *t*(106) = 0.234, *p* = 0.407. No interactions between condition and gender on support for conciliatory and aggressive statements were observed.

### Negative Emotions as Mediators of the Effect of Reappraisal on Support for Aggressive and Conciliatory Statements

Halperin et al. (2013) found that global score on negative emotions (i.e., PANAS Negative) and anger mediated the effect of reappraisal on support for conciliatory and aggressive statements. Here we tested whether these measures had the same mediating role. In addition, based on the reports that the campaign against the peace accord between government and FARC was successful by triggering fear in the population (El Espectador, 2016; El País, 2016), we assessed if this emotion had a mediating effect between reappraisal and support for conciliatory and aggressive statements.

All procedures were conducted using Model 4 of the SPSS macro PROCESS (Hayes, 2013) and 5,000 bootstrap resamples. We followed Hayes and Rockwood’s (2016) approach to determine whether a predicted variable M mediated the effect of an independent variable (X) on a dependent variable (Y). To that aim, we estimated the indirect effect of X (e.g., reappraisal) on Y (e.g., support for aggressive statements) operating through M (e.g., anger), and conducted an inference about that effect using a 95% bootstrap confidence interval (CI). A resulting CI that was entirely above or below zero (i.e., did not include zero) was considered evidence of a mediation effect, whereas a CI that included zero indicated no mediated effect of X on Y through M. Though in this approach the significance of the effects of X on M (e.g., reappraisal on anger) and M on Y (e.g., anger on support for aggressive statements) is not relevant in establishing mediation, as recommended by Hayes and Rockwood (2016) we included information on these effects to supplement the analysis – e.g., checking the consistency of the signs of the X-M and M-Y relationships with the predicted indirect effect.

#### The Mediating Effect of Negative Emotions on Support for Aggressive Statements

The prediction that negative emotions mediated the relationship between reappraisal and support for aggressive statements was confirmed with the observation that the indirect effect of reappraisal on support for aggressive statements through negative emotions was significant [95% CI did not include zero; (−0.354, −0.001)]. The signs of the associations between reappraisal and negative emotions (negative; *B* = −3.72, *p* = 0.016), and between negative emotions and support for aggressive statements (positive, *B* = 0.03, *p* = 0.05) were consistent with the predicted mediation effect.

We tested whether fear mediated the relationship between reappraisal and support for aggressive statements. The test of the indirect effect of reappraisal on support for aggressive statements through fear was significant [95% CI did not include zero; (−0.333, −0.002)], which confirmed that fear mediated the effects of reappraisal on support for aggressive statements. The signs of the associations between reappraisal and fear (negative, *B* = −0.43, *p* = 0.058), and between fear and support for aggressive statements (positive, *B* = 0.26, *p* = 0.011) were consistent with the predicted mediation effect.

Following Halperin et al. (2013), we tested whether anger mediated the relationship between reappraisal and support for negative statement. We found no significant indirect effect of reappraisal on support for aggressive statements through anger [95% CI did include zero; (−0.181, 0.056)].

#### The Mediating Effect of Negative Emotions on Support for Conciliatory Statements

The test for the indirect effect of reappraisal on support for conciliatory statements through negative emotions was significant [95% CI did not include zero; (0.01, 0.20)]; thus, results indicated that negative emotions do mediate the relationship between reappraisal and support for conciliatory statements. The signs of the associations between reappraisal and participants’ negative emotions (negative, *B* = −3.72, *p* = 0.016), and between negative emotions and support for conciliatory statements (negative, *B* = −1.69, *p* = 0.094) were consistent with the predicted mediation effect.

Regarding fear, the test for an indirect effect of reappraisal on support for conciliatory statements through fear was significant [95% CI did not include zero; (0.001, 0.229)], thus indicating that fear mediated the effects of reappraisal on support for conciliatory statements. The signs of the associations between reappraisal and fear (negative, *B* = −0.43, *p* = 0.058), and between fear and support for conciliatory statements (negative, *B* = −0.18, *p* = 0.015) were consistent with the predicted mediation effect.

Similar to Halperin et al. (2013), we tested whether anger mediated the relationship between reappraisal and support for conciliatory statements. We found that the indirect effect of reappraisal on support for conciliatory statements through anger was significant [95% CI did not include zero; (0.002, 0.189)], thus confirming the predicted mediating role of anger on the relationship between reappraisal and support for conciliatory statements. The signs of the associations between reappraisal and anger (negative, *B* = −0.44, *p* = 0.066), and between anger and support for conciliatory statements (negative, *B* = −0.13, *p* = 0.057) were consisted with the predicted mediation effect.

### Social Desirability

A two-tailed independent-samples *t* test on the Marlowe–Crowne Social Desirability Scale scores (Crowne and Marlowe, 1960 – adapted to Spanish by Ferrando and Chico, 2000) indicated no significant differences between reappraisal (*M* = 17.94, *SD* = 5.40) and control groups (*M* = 17.54, *SD* = 5.25), *t*(106) = 0.397, *p* = 0.692. The fact that these scores were between the average reported in Spanish (Ferrando and Chico, 2000) and Mexican (Lara-Cantú, 1990) populations (*M*_Spanish_ = 15.83, *SD* = 5.15; *M*_Mexican_ = 19.10, *SD* = 5.57) indicated that participants in the present study showed an average degree of concern for the social desirability of their responses.

## Discussion

The present study tested whether the effects of reappraisal training reported by Halperin et al. (2013) could be reproduced in the context of the armed conflict between the Colombian government and FARC. Reappraisal was expected to decrease the intensity of negative emotions evoked by anger-inducing information related to FARC’s violent actions, and produce more support for conciliatory statements and less support for aggressive statements. Also, we expected that negative emotions would mediate the relationship between reappraisal and support for conciliatory and aggressive statements.

Reappraisal training reduced the levels of negative emotions – anger, irritability, fear, and uneasiness – reported by the participants, with moderate-to-high effect magnitudes. It also significantly increased participants’ support for conciliatory statements related to the peace accord signed between FARC and the Colombian government (Colombian Government, 2016a), which was voted on a national referendum few days after the present experiment took place (e.g., acceptance of FARC’s members’ reincorporation to civil life). Also aligned with the findings reported by Halperin et al. (2013), our results indicated that negative emotions mediated the effects of reappraisal on the participants’ support for aggressive and conciliatory statements.

These findings add to those of previous studies (Gross et al., 2013; Halperin et al., 2013; Halperin, 2014; Rosler et al., 2015; Čehajić-Clancy et al., 2016; Gutentag et al., 2016) that support the notion that negative emotional experiences related to intractable conflicts limit attempts toward peaceful resolution of these conflicts (Gross et al., 2013; Halperin, 2014; Halperin and Pliskin, 2015). The replication of most of the effects observed in previous studies related to the Israeli–Palestinian conflict (Halperin and Gross, 2011; Halperin et al., 2013) suggest a high degree of generality of these phenomena, especially considering the differences between the conflicts approached, e.g., the Colombian conflict is internal, has entailed both state and non-state violence, several actors have been involved (other guerrillas, paramilitary groups, and drug cartels), and diverse types of violence were implemented on the civilian population, including terrorist acts, kidnapping, massacres, landmines, forced recruitment, forced displacement, disappearances, and selective executions (Historical Memory Group, 2016; Unidad para las Victimas, 2017). This aspect shows promise for further research that not only continue testing the generality of these effects on other types of intergroup conflicts across the world, but also attempt their application to generate socially relevant behavioral change (e.g., voting, donating, or volunteering). Also, related studies could explore the specific pathways through which these effects manifest depending on contextual factors of the conflict – e.g., why fear, and not anger, showed more evidence of mediating the relationship between reappraisal and support for conciliatory and aggressive statements in the Colombian context.

### Limitations and Further Research

The fact that we could only obtain evidence of an indirect effect of reappraisal on aggressive statements against FARC may relate to the characteristics of the texts that were presented to the participants—i.e., excerpts from the peace accord signed between FARC and Colombian government and aggressive statements from national newspapers. For instance, Correa et al. (2018) found that linguistic aspects of the Colombian government-FARC peace accord made its legibility very low, which adds to the more general finding that legal and political texts are not easily comprehended by lay persons (Charrow and Charrow, 1979; Jones et al., 2012; Mandic et al., 2012). Pilot testing of the different statements used in the present study was not possible due to the close date established for the referendum on the signed peace accord (less than 2 months), which may have resulted in the moderate levels of internal consistency observed for both sets of statements (α_conciliatory_ = 0.65; α_aggressive_ = 0.68). It is recommended that further research addresses these issues, including securing the legibility of the textual stimuli prior to their presentation to the participants (e.g., using data mining to identify the ideal or especially difficult fragments in a given text—Correa et al., 2018—and conducting pilot tests).

Another potential factor that may explain the limited effect of reappraisal on the participants’ attitudes toward the aggressive statements, is the fact that support for these ideas was overall low (scores typically ranged between 2 and 3 in the 6-pt Likert scale, irrespective of group assignment). This floor effect may have resulted from having chosen participants from a target population that was reported to be in favor of the peace accord (urban young adults; Caracol Radio, 2016), and thus against actions that may have compromised it. In addition, different to Halperin et al.’s (2013) experiment, which had a clearly identifiable outgroup, in the present study some participants may not have perceived FARC as an outgroup. Accordingly, further research could test the effects of reappraisal on populations with a different relationship to the conflict and its actors —e.g., victims and members of the armed forces—and thus other attitudes toward the peace process.

The assessment of changes in emotional states is an aspect that needs to be considered for future research. Same as in Halperin et al.’s (2013) study, participants’ emotions were only measured after receiving the cognitive reappraisal training (or control instructions) and the anger-inducing information. This limited the assessment of the effect of reappraisal, as participants’ emotional state at baseline was unknown. Though the strategy of randomly assigning participants to the reappraisal condition provided some degree of control over this potential confound, future studies could implement a pre and post intervention measurement.

Regarding the limited mediational role of anger, which contrasted with Halperin et al.’s (2013) findings, it seems plausible that this outcome relates to the overall low emotional level reported by the participants (below theoretical mean). This may have resulted from exclusion of participants that experienced traumatic events and/or the fact that Colombian young urban population characterizes for absence of strong emotions related to conflict-related information. Ongoing research in our lab is precisely testing the latter notion (Hurtado-Parrado et al., unpublished).

Lastly, the results of the national referendum on the peace accord (50.02% of the votes were against it) indicated a highly polarized public opinion across the Colombian population. This outcome suggests other lines of research that could entail (a) testing whether the effects reported in the present study could be reproduced in the population that voted against the accord, and (b) analyzing the contents and effects of the controversial campaign ran by Colombian opposition party (Centro Democrático) against the accord, which apparently was successful by triggering negative emotions in the voters (e.g., by distorting some of the contents of the accord regarding impunity for the FARC members and the negative economic and political implications of accepting the accord – El Espectador, 2016; El País, 2016). Exploring the role of fear in this context seems particularly relevant, since it was seemingly triggered by the opposition campaign and our data indicates a mediating role for supporting conciliatory and aggressive statements.

## Conclusion

Our findings add to those of previous research in showing the potential that resides in efforts to constructively cope with negative emotions that emerge during intractable conflicts. Future research should not only address the limitations of the present study, but also extend it by testing the long-term effects of the reappraisal intervention and its potential influence on actual political behavior (e.g., voting, donating money, or volunteering) and guiding public policy on areas affected by conflict. This is specially relevant in places in which efforts toward conflict resolution are currently being undertaken—as is the Colombian case.

Lastly, the positive effects of cognitive reappraisal training show promise to enhance the processes entailed in the development of peace cultures (Bar-Tal, 2000). This is particularly the case for the Colombian context, in which the much-needed processes of forgiveness and reconciliation seem heavily affected by negative emotions (e.g., feelings of hatred or distrust associated to a construed image of an enemy – Alzate et al., 2009; López-López et al., 2013, 2018; Cortés et al., 2015).

## Ethics Statement

This study was carried out in accordance with the ethical guidelines and recommendations of the American Psychological Association – APA (2002) and the Colombian Board of Psychologists (2016). All subjects gave written informed consent in accordance with the Declaration of Helsinki. The protocol was approved by the Research and Ethics Committee at Konrad Lorenz Fundación Universitaria.

## Author Contributions

CH-P, MS-P, MEH, AM, DG-V, JC, WL-L, and KH contributed to the conception, design, and implementation of the study. CH-P, MS-P, AM, DG-V, LV, and AC-C conducted the field work and organized the database. CH-P, MEH, and JR performed the statistical analysis. CH-P wrote the first draft of the manuscript. MEH, MS-P, WL-L revised and extended the manuscript. All authors contributed to manuscript revision, and read and approved the final version.

## Conflict of Interest Statement

The authors declare that the research was conducted in the absence of any commercial or financial relationships that could be construed as a potential conflict of interest.
