# Supplementary material for: Emotion Regulation and Attitudes Toward Conflict in Colombia: Effects of Reappraisal Training on Negative Emotions and Support for Conciliatory and Aggressive Statements
Source: Front Psychol. 2019 Apr 24;10:908. doi: 10.3389/fpsyg.2019.00908 (PMC6491871; doi:10.3389/fpsyg.2019.00908)
Supplement: Supplementary file 1 [file Table_1.DOCX]

**Appendix 1**

Conciliatory (C1-C3) and aggressive statements (A1-A3) to which participants of the study responded with their degree of support. Conciliatory statements consisted of excerpts copied verbatim from the peace accord signed between FARC and the Colombian government (Colombian Government, 2016a). Aggressive statements consisted of verbatim public statements made by political opponents of that peace agreement (e.g., statements in national newspapers).

| **Conciliatory Statements** |
| --- |
| C1. The Integral Rural Reform needs to be globally applied. Its execution will prioritize territories most affected by the conflict, misery, and governmental neglect via the development of programs with a local focus. These will be instruments of reconciliation in which all parties involved will contribute to the supreme right to peace, which is of mandatory enforcement. |
| C2. Peace building and consolidation, in the context of conflict ending between the government and FARC, require democratic opening that favor the appearance of new actors in the political stage. These forces will enrich the debate and favor pluralism, and thus the representation of different views and interests of the society. This process requires guarantees for participation and inclusion of these new actors in the political debate. |
| C3. Reincorporation of the FARC forces to the civil life will be an integral and sustainable process, exceptional and transitory, that will take into account the interests of the community and FARC, including their members and their families, and which will be oriented toward the regional strengthening of the social tissue. |
| **Aggressive Statements** |
| A1. The lack of justice, of adequate sanction to the criminals of the FARC, stimulates the emergence of new criminals; it does not favor reconciliation. Criminals are not punished, will be allowed to be politically elected, and will not be required to ask for forgiveness. They laugh at Colombians who are asking their recognition of regret. |
| A2. Although FARC announced one year ago a unilateral cease fire, they have not stopped dealing drugs or extorting. These are some of the criminal activities that they have never recognized, which affect the public order and the community. |
| A3. This peace process with FARC rewards terrorism with political eligibility, to which convicts, paramilitary, or dismissed politicians do not have the right to. |
